# Risk Perceptions and Behavior Among Hospitalized Patients With Smoking-Related Diseases

**Published:** 2009-09-15

**Authors:** Suzana E. Tanni, Nathalie Izumi Iritsu, Massaki Tani, Paula Angeleli Bueno de Camargo, Marina Gonçalves Elias Sampaio, Ilda Godoy, Irma Godoy

**Affiliations:** Botucatu Medical School, São Paulo State University, São Paulo, Brazil; Botucatu Medical School, São Paulo State University, São Paulo, Brazil; Botucatu Medical School, São Paulo State University, São Paulo, Brazil; Botucatu Medical School, São Paulo State University, São Paulo, Brazil; Botucatu Medical School, São Paulo State University, São Paulo, Brazil; Botucatu Medical School, São Paulo State University, São Paulo, Brazil; Botucatu Medical School, São Paulo State University, São Paulo, Brazil

## To the Editor:

Evidence in favor of implementing smoking cessation measures is strong, and such measures are critical for improving health outcomes among smokers and others who are exposed to secondhand smoke (1,2). Although some studies have evaluated the perception of the association between tobacco use and tobacco-related diseases (3), they have not, to our knowledge, investigated risk perceptions among patients who are hospitalized with smoking-related diseases. We surveyed a sample of such patients to evaluate their perceived risk and the potential for cessation interventions in this population.

We performed a cross-sectional analysis of 348 people who were smokers or former smokers and who were admitted to the University Hospital of Botucatu Medical School, São Paulo, Brazil, with tobacco-related diseases, from August 2007 through January 2008. We defined "tobacco-related disease" according to Brazilian Thoracic Society and World Health Organization guidelines, as follows: chronic obstructive pulmonary disease, interstitial lung disease, cerebral stroke, ischemic heart disease, venous thromboembolism, gastritis, gastric or duodenal ulcer, premature labor, and cancer (lung, oral, larynges, esophagus, gastric, pancreas, colon/rectal, bladder, kidney, breast, and uterus) (1,4). Exclusion criteria were patients who were in unstable condition (undergoing mechanical or noninvasive ventilation, with an unstable hemodynamic condition, or with alterations in mental status) or who were unable to answer or understand the survey questions. Participants gave written informed consent, and the hospital research ethics committee approved the study. We collected data on patient characteristics, knowledge about smoking-related diseases, smoking history, and smoking cessation treatment prescribed by health care professionals.

Most patients with smoking-related diseases were male (69%) and had fewer than 4 years of formal education (70%). The leading causes of hospitalization were cardiovascular disease (48%), cancer (20%), and respiratory diseases (12%). A substantial number of respondents (41%) were current smokers at the time of the hospitalization. This prevalence of smoking is much higher than that observed for the adult Brazilian population (10%-21%) (5). Exposure to secondhand smoke at home or work was reported by 85% of respondents. Patients reported that they had initiated smoking at a young age (mean 15 y), and 53% of the current smokers showed moderate-to-high nicotine dependence, according to the Fagerström score (6); 67% of the current smokers reported smoking the first cigarette within 30 minutes of waking up.

A high percentage of patients (65%) with smoking-related disease said they did not know about the association between smoking and their disease. However, 92% of respondents said they had been questioned by a health professional about their smoking status. More than 80% of respondents reported that they had received only brief counseling to stop smoking during the course of their disease, including the present hospitalization, and only 7% of the respondents had received specific smoking cessation treatment with counseling and medication.

Our findings suggest that hospitalization could be the ideal time to introduce guideline-based smoking cessation interventions including behavior counseling and pharmacologic treatment. These moments are windows of opportunity to improve patients' understanding of the relationship between smoking and their disease and to help them find smoking cessation treatment and follow-up after hospital discharge (7). Most patients (72%) were aware of smoking cessation methods, including those not recommended by the most recent guidelines, such as aversive technique or hypnosis (2,4). However, only 31% of patients reported knowing about the existence of specialized services for smoking cessation.

Limitations of our study include not taking into consideration the perspective of health care professionals and not identifying factors that may predict differences in risk perception and behavior between patients. We also did not investigate patients' reasons for not quitting when advised to do so. Therefore, we cannot exclude the possibility that the patients did not remember or did not comply with physician counseling or prescriptions.

Our findings of poor knowledge about the relationship between smoking and disease and the low rate of smoking cessation treatment reflect international problems (3,7). Training health professionals to adhere to guidelines by counseling their smoking patients more comprehensively, and implementing smoking cessation treatment among smoking patients who are hospitalized with smoking-related diseases, are effective tools to improve their health outcomes. Although costly, treatments for improving smokers' health outcomes are effective and they have been adopted, but the less expensive approach of smoking cessation treatment has been neglected.
